# Research on fertilization decision method for rice tillering stage based on the coupling of UAV hyperspectral remote sensing and WOFOST

**DOI:** 10.3389/fpls.2024.1405239

**Published:** 2024-06-07

**Authors:** Shilong Li, Zhongyu Jin, Juchi Bai, Shuang Xiang, Chenyi Xu, Fenghua Yu

**Affiliations:** ^1^ College of Information and Electrical Engineering, Shenyang Agricultural University, Shenyang, China; ^2^ National Digital Agriculture Sub-center of Innovation (Northeast Region), Shenyang, China; ^3^ Key Laboratory of Intelligent Agriculture in Liaoning Province, Shenyang, China

**Keywords:** unmanned aerial vehicle (UAV), hyperspectral, crop growth models, feature selection, data assimilation, rice fertilization

## Abstract

**Introduction:**

The use of chemical fertilizers in rice field management directly affects rice yield. Traditional rice cultivation often relies on the experience of farmers to develop fertilization plans, which cannot be adjusted according to the fertilizer requirements of rice. At present, agricultural drones are widely used for early monitoring of rice, but due to their lack of rationality, they cannot directly guide fertilization. How to accurately apply nitrogen fertilizer during the tillering stage to stabilize rice yield is an urgent problem to be solved in the current large-scale rice production process.

**Methods:**

WOFOST is a highly mechanistic crop growth model that can effectively simulate the effects of fertilization on rice growth and development. However, due to its lack of spatial heterogeneity, its ability to simulate crop growth at the field level is weak. This study is based on UAV remote sensing to obtain hyperspectral data of rice canopy and assimilation with the WOFOST crop growth model, to study the decision-making method of nitrogen fertilizer application during the rice tillering stage. Extracting hyperspectral features of rice canopy using Continuous Projection Algorithm and constructing a hyperspectral inversion model for rice biomass based on Extreme Learning Machine. By using two data assimilation methods, Ensemble Kalman Filter and Four-Dimensional Variational, the inverted biomass of the rice biomass hyperspectral inversion model and the localized WOFOST crop growth model were assimilated, and the simulation results of the WOFOST model were corrected. With the average yield as the goal, use the WOFOST model to formulate fertilization decisions and create a fertilization prescription map to achieve precise fertilization during the tillering stage of rice.

**Results:**

The research results indicate that the training set *R^2^* and RMSE of the rice biomass hyperspectral inversion model are 0.953 and 0.076, respectively, while the testing set *R^2^* and RMSE are 0.914 and 0.110, respectively. When obtaining the same yield, the fertilization strategy based on the ENKF assimilation method applied less fertilizer, reducing 5.9% compared to the standard fertilization scheme.

**Discussion:**

This study enhances the rationality of unmanned aerial vehicle remote sensing machines through data assimilation, providing a new theoretical basis for the decision-making of rice fertilization.

## 1 Introduction

Rice is one of the world’s major food crops (Lv et al., 2018). Fertilizer, as an important component of rice field management, directly affects rice yield. Fertilization plans formulated based on the experience of farmers are prone to excessive fertilization, resulting in rice lodging or reduced rice yield due to pests and diseases. Agricultural drones have the advantages of high operational efficiency and precise spraying, and are currently widely used in rice monitoring and fertilization operations, providing new choices for rice field management.

During the growth process of rice, there are two peak periods of fertilizer demand, namely the tillering stage (Wang et al., 2021) and the booting stage (Lai and Lin, 2021). The tillering stage is the first peak fertilizer requirement for rice, and the tillering ability of rice plays a decisive role in the later stage of rice panicle formation and growth rate. The growth status of rice is an important basis for making precise fertilization decisions. Traditional methods of collecting field samples to obtain the growth status of rice are costly and time-consuming, making it difficult to meet the requirements of precise fertilization. Obtaining spectral data through unmanned aerial vehicle spectroscopy equipment (Liu et al., 2023a) has the characteristics of low cost and fast acquisition speed. At present, relevant research mainly constructs mathematical models of spectral data and physiological parameters of rice through multispectral and hyperspectral data, achieving rapid inversion of rice growth and development, and providing guidance for nitrogen fertilizer management plans (Li et al., 2021; Lu et al., 2021; Jia et al., 2022; Xu et al., 2023). Wang et al. constructed a nitrogen concentration model for rice stems and leaves using hyperspectral data from drones, providing a method for estimating nitrogen concentration in rice stems and leaf organs (Wang et al., 2023). Luo et al. used drone multispectral images to construct a rice yield estimation model for the heading stage, filling the gap in traditional ground measurements that were only applicable to rice yield estimation during the booting stage (Luo et al., 2022). Chen et al. used drones to obtain multispectral data on rice canopy and established a quantitative inversion model for monitoring the suitable harvest period of rice, filling the research gap of combining harvest yield and spectral characteristics (Cong et al., 2022). Due to the influence of various factors such as variety, climate, soil, and diseases on crop growth and development (Challinor et al., 2005; He et al., 2019; Deepika and Kaliraj, 2021), it is a complex process to determine the specific development status and nitrogen fertilizer demand of crops based on the obtained physiological parameters. In summary, most current research focuses on quickly inverting the physicochemical parameters of rice growth status in a data-driven manner, but due to its lack of mechanistic mechanisms, it cannot directly guide fertilization.

The crop growth model is a highly mechanistic model that takes into account the temporal characteristics of crop growth and development and the instantaneous characteristics of physiological parameter acquisition. By using data assimilation methods to combine the obtained physiological parameters with the crop growth model, the growth process simulation of the crop growth model is corrected through physiological parameters, Realizing real-time correction of crop growth and development and nitrogen fertilizer management plans has become a feasible approach for generating precise fertilization plans. The crop growth model combines multiple disciplines such as crop physiology, meteorology, soil science, hydrology, etc. Based on crop physiology theory, a dynamic model of crop growth and development under the influence of environmental factors such as soil, water, and fertilizer is constructed, and a large amount of field measurement data is used to simulate the crop growth process. Based on different theoretical foundations and research objects, commonly used crop growth models currently include APSIM (Kumar et al., 2021), DSSAT (Ma et al., 2020), ORYZA2000 (Lu et al., 2019), WOFOST (Ju et al., 2023), etc. Data assimilation was first proposed by Charney (Charney et al., 1969) and was initially used in fields such as numerical weather forecasting and ocean circulation models. Because the essence of data assimilation is to integrate new observation data during the dynamic operation of the data model, achieve real-time correction of model trajectory, and improve the estimation accuracy of the model, data assimilation has been widely applied in fields such as ocean monitoring (Tang et al., 2020), soil monitoring (Zhou et al., 2022), and crop growth simulation (Dong et al., 2013). At present, there is not enough research in the field of crop growth model data assimilation. Chen et al. studied the potential of assimilating LAI for early season crop yield prediction and obtained wheat yield prediction (Chen and Tao, 2020).Li et al. combined the CERES-Wheat model with remote sensing data using an integrated Kaiman filter to optimize the key wheat parameter LAI (Li et al., 2011). Pique et al. assimilated satellite remote sensing data into crop models to estimate the daily carbon dioxide flux and carbon budget components of wheat (Pique et al., 2020).

**Figure 1:**
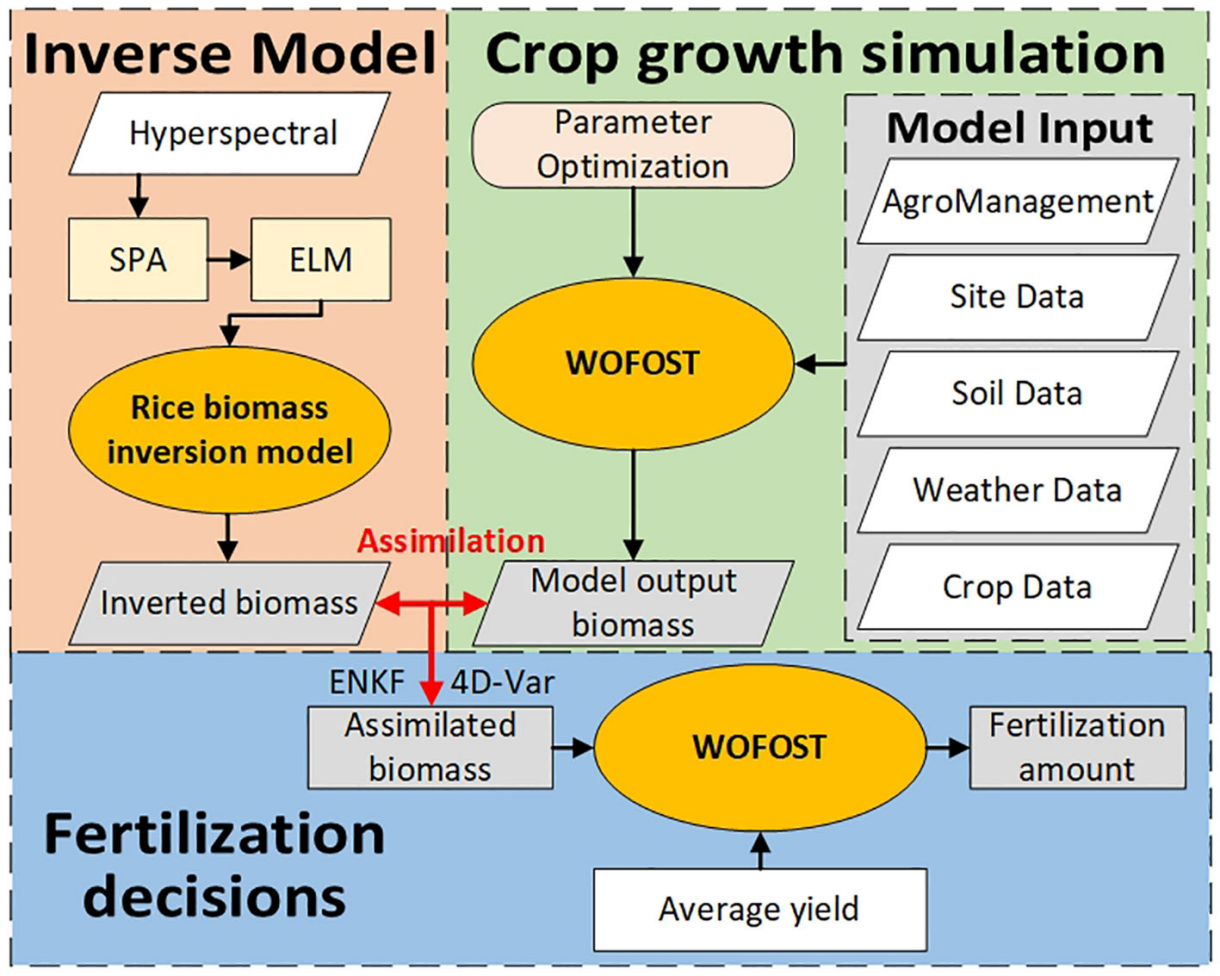
Overview of the research.

In summary, it is feasible to combine hyperspectral inversion technology with data assimilation methods and crop growth models. However, current research focuses more on improving the accuracy of growth models and yield prediction, and does not link this method with precision fertilization. As shown in **Figure 1**, this study first constructed a rice biomass hyperspectral inversion model using unmanned aerial vehicle (UAV) rice canopy hyperspectral data and rice biomass data to achieve rapid acquisition of rice biomass data; Then, using rice biomass as the assimilation parameter, the ensemble Kalman filter algorithm (ENKF) and four-dimensional variational algorithm (4D-Var) are used to simulate the rice growth process based on data assimilation; Finally, with the average yield as the target, the fertilization plan obtained from WOFOST will be used as the final fertilization plan. By constructing a fertilization prescription chart, variable fertilization of rice will be achieved, and the fertilization plan will be evaluated based on the final yield.

## 2 Materials and methods

### 2.1 Experimental design

The experimental site is located at the Precision Agriculture Aviation Research Base of Shenyang Agricultural University in Gengzhuang Town, Haicheng City, Liaoning Province (40° 58 ‘45.39 “N, 122° 43’ 30.00” E). The experimental site is in a warm temperate monsoon climate zone, with a mild climate throughout the area, an average annual temperature of 10.4 °C, and a rainfall of 721.3 millimeters. The experimental variety is the mid to late-maturing japonica rice “Shennong 9816” widely planted in the Liaoning region. The variety has a growth period of 157 days and an average yield of 9000 kg/ha.

The data collection for inverting biomass modeling and localizing crop growth models was conducted from June to September 2020. Rice canopy hyperspectral reflectance measurements were conducted once a week throughout the entire growth period of rice, and field experiments were conducted to measure LAI. At the same time, four samples were taken from each plot to measure biomass.

**Figure 2:**
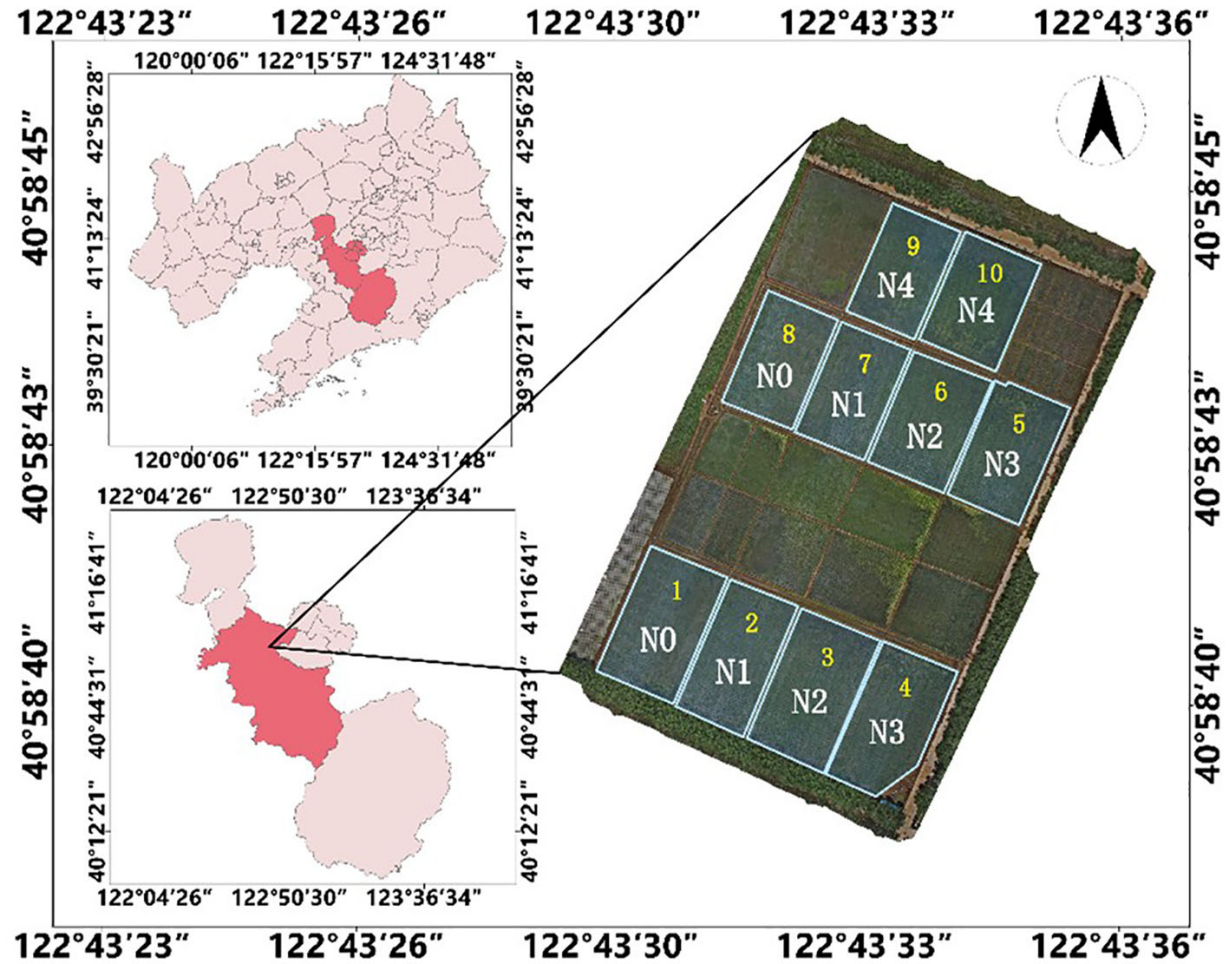
Test plot design.

The topdressing experiment was conducted from June to September 2021, and the experimental community was designed with 5 nitrogen fertilizer gradient treatments (**Figure 2**), namely N0, N1, N2, N3, and N4; Each community is separated by a field ridge. Among them, N0 is the control group, that is, no basal fertilizer is applied; N3 is the local standard nitrogen fertilizer application level, with a nitrogen fertilizer application rate of 200 kg/ha. N1 and N2 are low nitrogen fertilization levels, with application rates of 100 kg/ha and 150 kg/ha, respectively; N4 is a high nitrogen fertilization level, with an application rate of 250 kg/ha; The application of phosphorus and potassium fertilizers is carried out according to local standard application rates, with a standard application rate of 144 kg/ha for phosphorus fertilizer and 192 kg/ha for potassium fertilizer. The base fertilizer in each experimental community is applied at 50% of different nitrogen fertilizer gradients, tillering fertilizer is applied according to crop growth variables, panicle fertilizer is applied at 20% of the local standard nitrogen base fertilizer application level, and other field management is carried out at the local normal level.

### 2.2 Data acquisition

#### 2.2.1 Drone rice canopy hyperspectral acquisition

This study used the M600 PRO six-rotor unmanned aerial vehicle from Shenzhen DJI Innovation Company as the flight platform, and the hyperspectral imager used the GaiaSky-mini built-in push broom airborne hyperspectral imaging system from Sichuan Shuangli Hepu Company. The hyperspectral band range is 400–1000 nm, with a resolution of 3.5 nm and 170 effective bands. The acquisition time for a single image is 15 seconds, the frame rate is 162 frames/s, and the drone flies at an altitude of 100m and a spatial resolution of 7cm. This study selected the time for collecting hyperspectral data from drones between 12:00–14:00, and performed dark current correction and whiteboard correction on the hyperspectral imager before takeoff. At the same time, place a 1.5m * 1.5m diffuse reflection plate with a reflectance of 60% in each hyperspectral acquisition area for later reflection data correction. Using the SpectraVIEW software paired with the airborne hyperspectral imager, preprocess the obtained unmanned aerial vehicle hyperspectral remote sensing images with lens, uniformity, reflectance, etc., and finally obtain the rice canopy hyperspectral reflectance image.

#### 2.2.2 Obtaining agricultural parameters of rice

The leaf area index (LAI) of the rice canopy throughout the entire growth period in the experimental community is an important target result for parameter calibration in the WOFOST model. When measuring the LAI of the entire growth period, the LAI-2200C plant canopy analyzer (Wang et al., 2020) was used to collect data. When collecting, place the fisheye lens above and below the canopy to measure the radiation value (A value) without obstruction and the radiation value (B value) below the canopy. To ensure the accuracy of measurement results, the average LAI of three points collected from each experimental field is taken as the measured LAI of the experimental community.

This study requires obtaining aboveground dry matter weight for inversion modeling of rice biomass. When measuring the dry matter weight of the sample on the ground, destructive sampling is first conducted on the rice in each community. The sample is taken to the laboratory, and then placed in an oven to kill at 105 °C for 30 minutes. After that, the sample is dried at 80 °C to a constant weight; Afterwards, measure the dry matter mass of the dried sample. The obtained agronomic parameters of rice are shown in **Table 1**.

**Table 1:** Agricultural parameter statistical data.

	Min	Max	Average	Standard deviation
Biomass(t/ha)	0.009	1.34	0.37	0.32
Leaf area index	0.44	5.36	3.82	1.57

### 2.3 Construction of a hyperspectral inversion model for rice biomass

**Figure 3:**
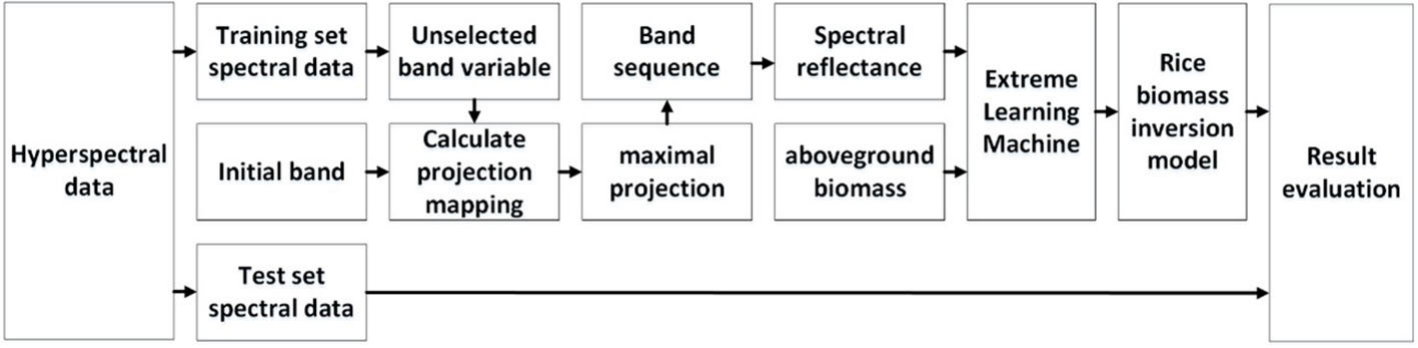
Hyperspectral inversion method for rice biomass.

To modify the growth simulation process of the WOFOST model by assimilating rice biomass data, it is necessary to quickly obtain rice field biomass data. This study constructs a hyperspectral inversion model for rice biomass using rice canopy spectral data to achieve rapid acquisition of rice biomass data. The model extracts hyperspectral features of the rice canopy using a Continuous Projections Algorithm (SPA), and constructs a rice biomass inversion model based on an Extreme Learning Machine (ELM). The construction process of the rice biomass inversion model is shown in **Figure 3**.

#### 2.3.1 Selection of hyperspectral features in rice canopy

Due to the high dimensionality of rice canopy hyperspectral data obtained by drones, it contains a large amount of redundant information. Directly using raw data for inversion modeling can significantly improve the computational time of the model and reduce its inversion accuracy. Continuous Projections Algorithm (SPA) is a forward variable selection algorithm that minimizes vector space collinearity and is commonly used for screening spectral feature variables (Zhang et al., 2023). This study uses SPA to extract features from hyperspectral data, and the extracted spectral reflectance of the feature bands is used as input for subsequent inversion models. SPA starts by selecting a wavelength and then merges another variable at each iteration until a specific number of defined variables are completed. It is used to select the wavelength with the least redundant spectral information to solve the collinearity problem. The core formula of SPA is as follows.

(1) Initialize:


$$n=1,x_{j}\in X_{j},j=1\cdots ,J$$

(2) Determine variables for unselected bands:


$$S=\left\{X_{i},1\leq i\leq J,i\notin \left\{k\left(0\right),\cdots ,k\left(N-1\right)\right\}\right\}$$

(3) Calculate the projection mapping for unselected and initialized bands:


$$Px_{j}=x_{j}-\left(x_{j}^{T}x_{i}\right)x_{i}{\left(x_{i}^{T}x_{i}\right)}^{-1},x_{i}\in S$$

(4) Determine maximum projection:


$$k\left(n\right)=max\left(\left|\left|Px_{j}\right|\right|\right),x_{j}\in S$$

(5) Assignment:


$$x_{j}=Px_{j},\;j\in S$$

(6) Determine the selection of band sequence:


{k(n);n=0,⋯,N−1}

Among them, X is the spectrum, k(0) is the initial band, the number of bands to be extracted is N , the spectral matrix is column J , the unselected variable in the original matrix is S , and the orthogonal projection is $Px_{j}$ .

#### 2.3.2 Rice biomass inversion modeling

To achieve rapid inversion modeling of rice biomass data, this study takes the training set rice characteristic spectral reflectance and biomass data as inputs, constructs a rice biomass inversion model based on Extreme Learning Machine (ELM) (Deng et al., 2019), and inputs the test set spectral data into the model as result validation. ELM is a machine learning method based on Feedforward Neural Network (FNN) and its backpropagation algorithm improvement. Compared with shallow learning systems such as Support Vector Machines, ELM has more advantages in learning ability and generalization ability.

Before constructing the model, in order to reduce the risk of overfitting, enhance generalization ability, and avoid the impact of sample differences, the sample dataset composed of unmanned aerial vehicle canopy hyperspectral data and biomass obtained from field experiments was randomly divided into two groups in a ratio of 7:3 as training and testing sets for constructing biomass inversion models and model evaluation.

#### 2.3.3 Evaluation criteria for inversion results

For the inversion results of rice biomass inversion models, this study uses *R^2^* and Root Mean Square Error (RMSE) to determine the inversion accuracy. *R^2^*, also known as correlation index, is mainly used to describe the linear relationship between two variables and is usually used to determine the degree of fit of regression models. It is between 0 and 1, and the higher the *R^2^* value, the smaller the sum of squared residuals, and the better the fitting degree. It is used to measure the deviation between observed values and true values, and the calculation method is as follows:


$$R^{2}=\frac{ESS}{TSS}=1-\frac{RSS}{TSS}=\frac{\sum_{j=1}^{n}{\left(X_{pred,j}-\bar{X_{meas}}\right)}^{2}}{\sum_{j=1}^{n}{\left(X_{meas,j}-\bar{X_{meas}}\right)}^{2}}=1-\frac{\sum_{j=1}^{n}{\left(X_{meas,j}-X_{pred,j}\right)}^{2}}{\sum_{j=1}^{n}{\left(X_{meas,j}-\bar{X_{meas}}\right)}^{2}}$$

Among them, TSS is the sum of population squares, ESS is the sum of regression squares, RSS is the sum of residual squares, ${\text{X}}_{\text{pred}}$ is the predicted value, ${\text{X}}_{\text{meas}}$ is the observed value, $\bar{{\text{X}}_{\text{meas}}}$ is the average of the observed values, and n is the sample size.

RMSE, also known as root mean square error, is used to measure the average deviation between model predictions and observations. The smaller the RMSE, the better the regression performance of the model. The calculation method is as follows:


$$RMSE=\sqrt{\frac{\sum_{j=1}^{n}{\left(X_{pred,j}-X_{meas,j}\right)}^{2}}{n}}$$

Among them, ${\text{X}}_{\text{pred}}$ is the predicted value, ${\text{X}}_{\text{meas}}$ is the observed value, and n is the sample size.

### 2.4 Constructing rice fertilization decision based on WOFOST model data assimilation

**Figure 4:**
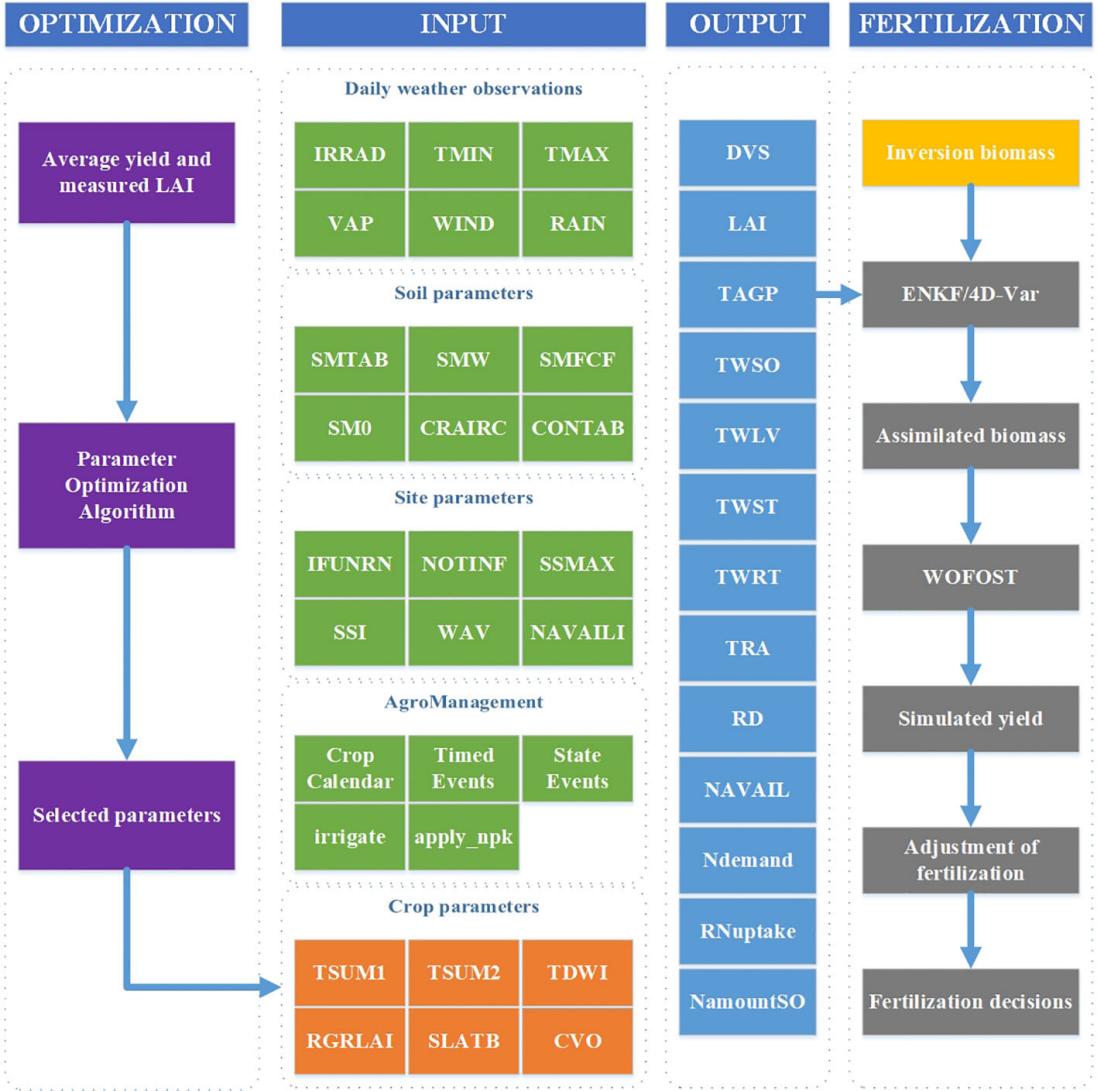
Fertilization decision making. Parameters of WOFOST were calibrated with average yield and measured LAI to localize the model. The model output biomass was assimilated with the inversion model output biomass to obtain the assimilated biomass, which was then used to formulate the fertilization decision.

This study corrected the WOFOST model by assimilating the rice biomass inversion results with the local model output biomass. The output yield of the modified model is the simulated yield under the current fertilization scheme in the experimental plot. Based on simulated yield, adjust the fertilization amount of the WOFOST model to achieve the goal of output yield approaching the average yield of the variety, formulate fertilization decisions, and construct a fertilization prescription map according to the fertilization decisions to achieve fertilization. The process diagram for making fertilizer application decisions is shown in **Figure 4**.

#### 2.4.1 Parameter acquisition and construction of WOFOST model

**Figure 5:**
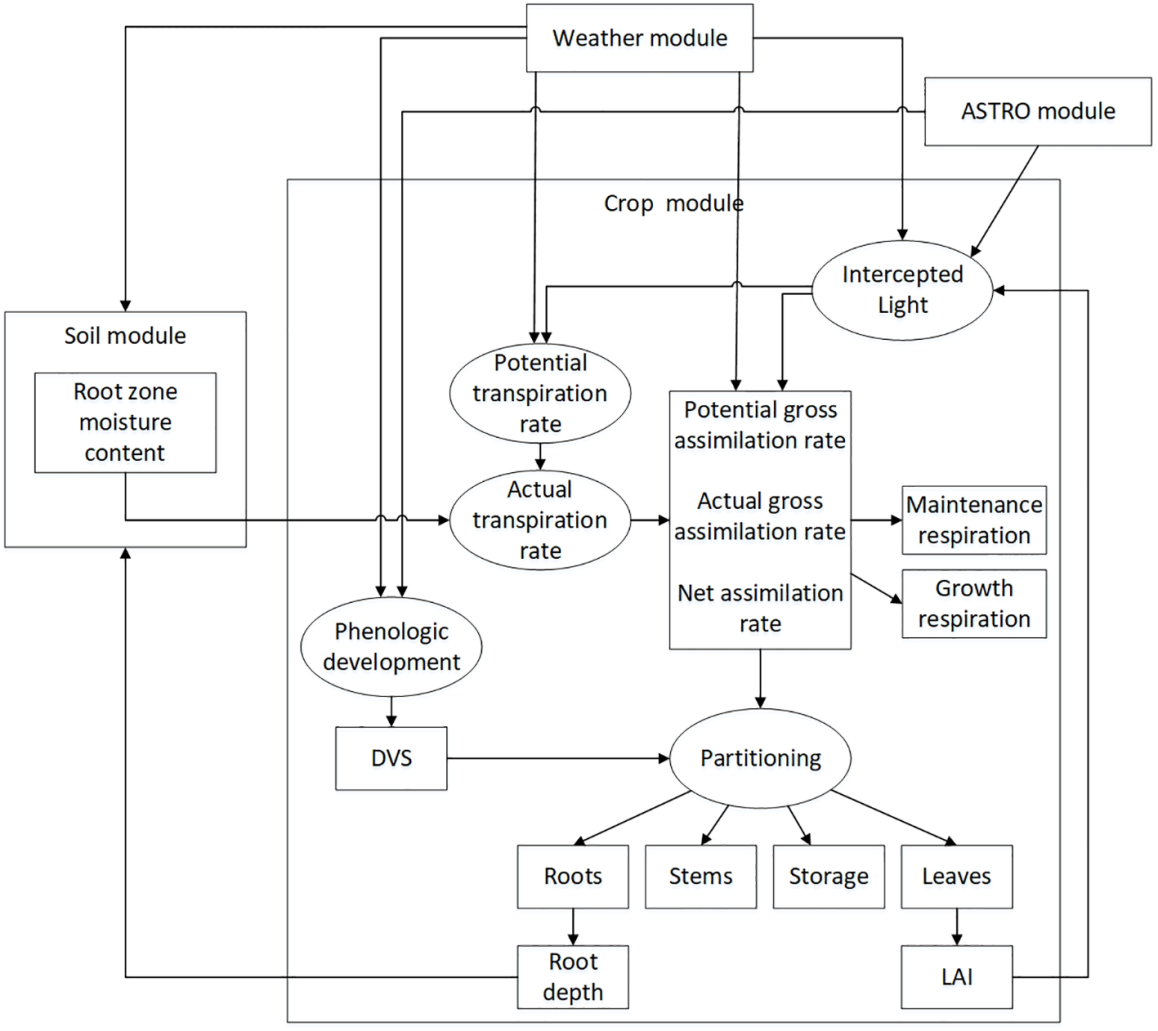
WOFOST model structure process (de Wit et al., 2019).

The WOFOST (World Food Studies) model is a single site based simulation system developed by the University of Wageningen in the Netherlands. It was originally developed to evaluate the production potential of tropical regions and the impact of meteorological and hydrological conditions on annual crops. Compared with traditional crop growth models, the WOFOST model focuses more on simulating annual field crops. Compared with the SUCROS model developed in the same “De Wit school”, the WOFOST model is more universal and only requires adjusting relevant crop parameters to simulate different crops such as rice (Zhou et al., 2018; He et al., 2019). WOFOST, as a mechanistic model, starts from the fundamental theories of crop physiology, ecology, physics, and other disciplines to model and simulate the physiological and physical processes of crop growth and development, which can provide more accurate predictions of crop growth, development, and yield. Based on simulating crop physiological processes, including CO_2_ assimilation, crop respiration, crop transpiration, and dry matter distribution (de Wit et al., 2019). This model can simulate crop growth with a time step of days and supports three modes: simulating potential, limiting water, and limiting nutrients. The WOFOST model is currently widely used in crop yield estimation (Liu et al., 2023b), analyzing the effects of fertilization (Tang et al., 2023) and irrigation (Amiri et al., 2022) factors on crop growth and development processes, and other fields. Its input parameters include agricultural management, crop parameters, soil parameters, scene parameters, weather parameters, etc. The main process of the WOFOST model is shown in **Figure 5**.

The traditional WOFOST model is written in FORTRAN language, but with the development of computer technology, FORTRAN language is difficult to integrate with tools such as databases and has poor flexibility. Therefore, researchers such as De Wit have re-implemented the WOFOST model using Python language (de Wit et al., 2019).PCSE (The Python Crop Simulation Environment) is a Python software package that includes models such as WOFOST and LINGRA (Dewenam et al., 2021). Compared to traditional WOFOST written using FORTRAN language or FORTRAN Simulation Environment (FSE), PCSE is more versatile. Currently, PCSE supports two versions of WOFOST models, WOFOST72 and WOFOST80. This study compared the effects of two versions of the WOFOST model on crop growth and development under different fertilization strategies, and selected a model version that is more suitable for simulating the impact of fertilization strategies on crop growth and development.

To simulate local crops using the WOFOST model, the first step is to conduct research on model localization. For easily obtainable parameters, this study will use some rice crop parameters, experimental field soil parameters, scene parameters, average weather conditions in the past three years, and crop sowing, irrigation, and fertilization information obtained through actual measurement methods to input into the model. Due to some crop parameters in the WOFOST model that cannot be calibrated through experiments, based on the average yield of this rice variety “Shennong 9816” and the measured leaf area index in the 2020 field experiment, parameter optimization algorithms were used to localize the model, and the accuracy of the parameter optimization results was determined by combining experimental data. Input the parameters obtained by localizing the above model into the WOFOST model, and simulate the output results of aboveground biomass, yield, fertilization amount, etc. based on the current production plan.

#### 2.4.2 Research methods for data assimilation

Single remote sensing data to guide fertilizer chasing has the characteristics of strong observation ability and real-time, but it lacks mechanism; the WOFOST model simulates the process of crop growth and development day by day from the perspective of crop growth mechanism, but a single model simulation lacks the simulation of uneven spatial distribution (heterogeneity). The coupling of four-dimensional variational algorithm (4D-Var) and ensemble Kalman filtering algorithm (ENKF) with localized WOFOST model output biomass results and remote sensing inversion biomass results can achieve complementary advantages and disadvantages, and calculate the simulated yield under current growth conditions.

Data assimilation is a method of continuously integrating external observation information in the dynamic operation simulation of numerical models to achieve automatic model adjustment. It is now widely used in weather forecasting and automotive navigation systems. According to the correlation mechanism between data assimilation algorithms and models, data assimilation methods can be divided into two categories: continuous data assimilation algorithms such as three-dimensional variational algorithms (Storto and Tveter, 2009), and four-dimensional variational algorithms (Kärcher et al., 2018), and sequential data assimilation algorithms such as ensemble Kalman filters (Pu and Hacker, 2009) and particle filters (Li et al., 2019).

The variational assimilation algorithm is a method that uses the principle of variation to globally adjust simulation results using all observed values. By minimizing the cost function, the analysis field is optimized in a statistical sense. The core idea is to transform the assimilation problem of observations into a variational problem with constraints, which are dynamic simulation equations. The main process of the four-dimensional variational algorithm (4D-Var) is as follows:

(1) State Vector:


$$x_{t+1}=m_{t}\left(x_{t}\right)$$

(2) Observations:


$$y_{t}=h_{t}\left(x_{t}\right)+\varepsilon_{t}$$

(3) Cost Function:


$$J_{\left(x_{0}\right)}=\frac{1}{2}{\left(x_{0}-x_{b}\right)}^{T}B^{-1}\left(x_{0}-x_{b}\right)+\frac{1}{2}\sum_{t=0}^{N}\left(h_{t}\left(x_{t}\right)-y_{t}\right)R_{t}^{-1}\left(h_{t}\left(x_{t}\right)-y_{t}\right)$$

$$J_{\left(x_{0}\right)}=\frac{1}{2}{\left(x_{0}-x_{b}\right)}^{T}B^{-1}\left(x_{0}-x_{b}\right)+\frac{1}{2}\sum_{t=0}^{N}{\left(h_{t}\left(m_{0\to t}\left(x_{0}\right)\right)-y_{t}\right)}^{T}R_{t}^{-1}\left(h_{t}\left(m_{0\to t}\left(x_{0}\right)\right)-y_{t}\right)$$

$$J_{\left(x_{0}\right)}=\frac{1}{2}{\left(x_{0}-x_{b}\right)}^{T}B^{-1}\left(x_{0}-x_{b}\right)+\frac{1}{2}{\left(\hat{h}\left(x_{0}\right)-\hat{y}\right)}^{T}R^{-1}\left(\hat{h}\left(x_{0}\right)-\hat{y}\right)$$

(4) Optimization:


$$\nabla J\left(x_{0}\right)=B^{-1}\left(x_{0}-x_{b}\right)+j{\left(\hat{h}\left(x_{0}\right)-\hat{y}\right)}^{T}R^{-1}\left(\hat{h}\left(x_{0}\right)-\hat{y}\right)$$

Among them, x and y are the model state prediction and observation values, respectively; $x_{0}$ and $x_{b}$ are the initial and background fields of the model state variables, while $x_{b}$ is a prior estimate of $x_{0}$ ; B and R are the covariance matrices of background error and observation error, respectively; $h_{t}$ is the observation operator; $y_{t}$ is the observation vector at time t ; $x_{t}$ is the predicted value of the model at time t ; J is the Jacobian matrix.

The Ensemble Kalman Filter (ENKF) algorithm based on statistical estimation theory solves the problem of nonlinear operators in a set form, and the extension method of the Ensemble Kalman Filter algorithm can also achieve ideal results under non-Gaussian conditions (Grooms, 2022). The working principle can be briefly summarized as follows: the simulation state advances over time until the observation data is analyzed, and the model state is adjusted based on the uncertainty of the model state. This will cause the simulation state to “jump” to a new state and continue to move forward. The Ensemble Kalman Filter algorithm mainly consists of two parts: prediction equation and update equation, and its main process is as follows:

(1) Variable prediction:


$$X_{k}^{F}=MX_{k-1}^{A}$$

(2) Prediction error analysis:


$$P_{k}^{F}H^{T}=\bar{\left(X_{k}^{F}-{\bar{X}}_{k}^{F}\right){\left(HX_{k}^{F}-H{\bar{X}}_{k}^{F}\right)}^{T}}$$

$$HP_{k}^{F}H^{T}=\bar{\left(HX_{k}^{F}-H{\bar{X}}_{k}^{F}\right){\left(HX_{k}^{F}-H{\bar{X}}_{k}^{F}\right)}^{T}}$$

(3) Calculate the gain matrix:


$$K_{k}=P_{k}^{F}H_{k}^{T}{\left[R_{k}+H_{k}P_{k}^{F}H_{k}^{T}\right]}^{-1}$$

(4) Update variables:


$$X_{k}^{A}=X_{k}^{F}+K_{k}\left[y_{k}-H_{k}X_{k}^{F}\right]$$

Among them, X is the set of model state variables with disturbances; M is the prediction matrix; $P^{F}$ is the covariance of the prediction field error; $P^{A}$ is the covariance of the analysis field error; F represents prediction; A represents analysis; K is the gain matrix; H is the observation operator; R is the covariance of observation error; y is a set of observations with perturbations.

This study used the four-dimensional variational algorithm and ensemble Kalman filtering algorithm to assimilate the inverted biomass with the output biomass of the localized model. The WOFOST model was modified, and the output yield of the modified model is the simulated yield under the current growth trend.

#### 2.4.3 Generate UAV operation prescription map

Based on the fertilization plans of each experimental plot, the required fertilization amount for rice tillering stage in each experimental plot is adjusted to make the simulated yield output of the WOFOST model modified by two assimilation algorithms approach the average yield of the rice variety. Divide each experimental plot into two equal parts, and apply two assimilation algorithms to correct the model. Output the required amount of fertilizer when the yield reaches the average yield, generate fertilizer decisions, and draw a fertilizer application prescription map for plant protection drones based on the amount of fertilizer. Guide the drones to carry out variable fertilizer application operations for rice.

## 3 Results and analysis

### 3.1 Hyperspectral inversion results of rice biomass

#### 3.1.1 Selection of hyperspectral features in rice canopy

**Figure 6:**
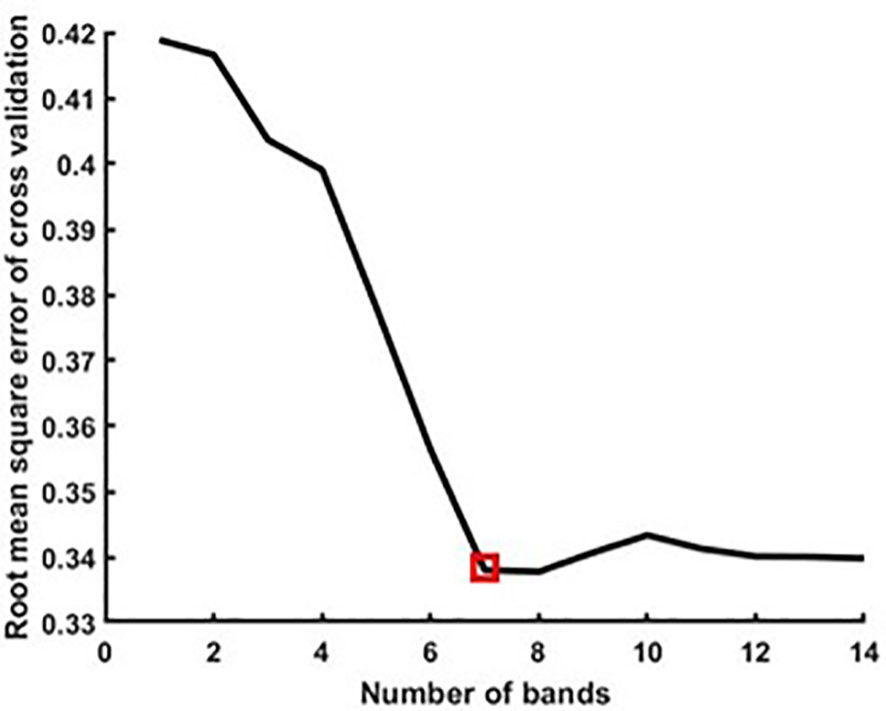
Selection results of SPA for hyperspectral characteristic bands of rice biomass.

This study used SPA for feature selection of rice canopy hyperspectral data, with a range of 5–30 bands. The selected bands were internally cross-validated using a correction set. Based on the root mean square error of cross-validation (RMSECV) of the validation results, the rice biomass hyperspectral feature bands were selected. The lowest RMSECV value is the optimal subset wavelength data. As shown in **Figure 6**, when using SPA for feature band screening, the overall RMSECV shows a downward trend as the number of screening variables increases. When RMSECV is at its lowest, 7 spectral characteristic bands were extracted, with wavelengths of 406, 418, 448, 613, 716, 763, and 919nm, respectively.

#### 3.1.2 Hyperspectral inversion of rice biomass

**Figure 7:**
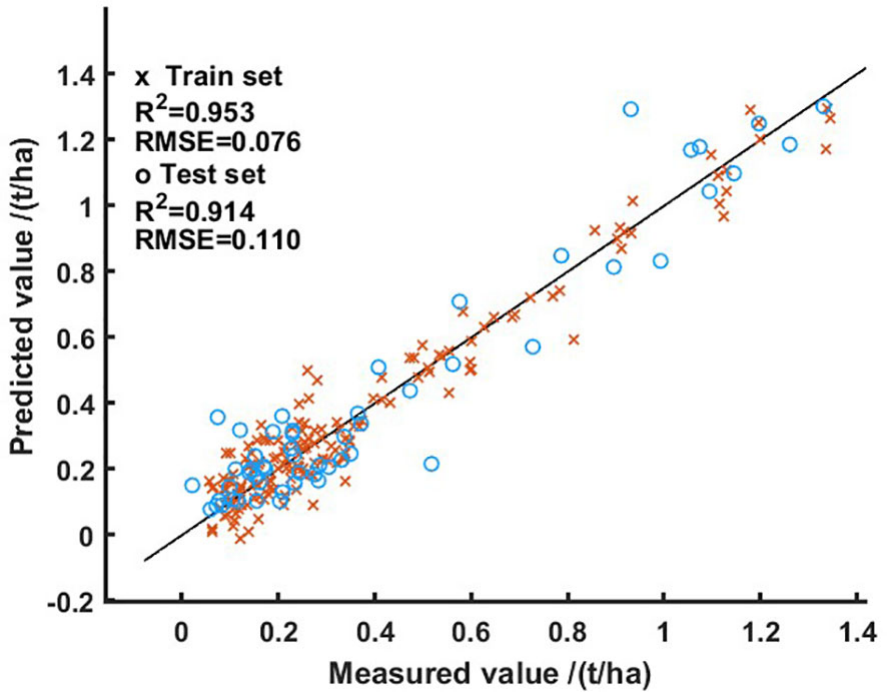
Results of rice hyperspectral biomass inversion model.

This study constructs a rice biomass inversion model based on the ELM model, using the spectral and biomass data extracted from the rice characteristic bands in section 3.1.1 as input. The inversion results of the model are shown in **Figure 7**. It can be seen from the results that the model has a high accuracy in inverting rice biomass, with training sets *R^2^* and RMSE of 0.953 and 0.076, respectively; The test sets *R^2^* and RMSE are 0.914 and 0.110, respectively. Overall, the rice biomass inversion model combining SPA feature selection and ELM can effectively estimate rice biomass information, providing data support for subsequent data assimilation modeling.

### 3.2 Simulation results of rice growth process based on WOFOST data assimilation

**Figure 8:**
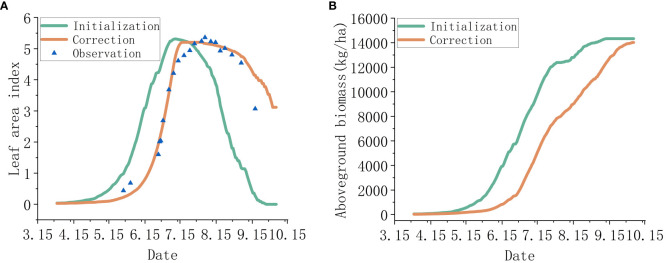
Localization results of the WOFOST model. Among them, panel **(A)** shows the localization process based on observed LAI, and panel **(B)** shows the comparison of biomass output of the model before and after localization.

This study obtained field data based on the parameter requirements of the WOFOST model, including some rice crop parameters, experimental field soil parameters, scene parameters, average weather conditions in the past three years, as well as crop sowing, irrigation, and fertilization. For parameters that are difficult to determine, parameter optimization algorithms were used to calibrate them in order of the growth period, measured leaf area index, and average yield of the studied crops. By using the WOFOST model to simulate the rice growth process, the localized WOFOST rice growth process was obtained as shown in **Figure 8**. **Figure 8A** shows the localization process of the model based on observed LAI, and **Figure 8B** shows the output biomass of the localized model. The output results of the model are consistent with the measured results, and the successful localization of the model simulates the growth and development of rice in the experimental field, laying a foundation for further research on data assimilation and guidance for top dressing.

**Figure 9:**
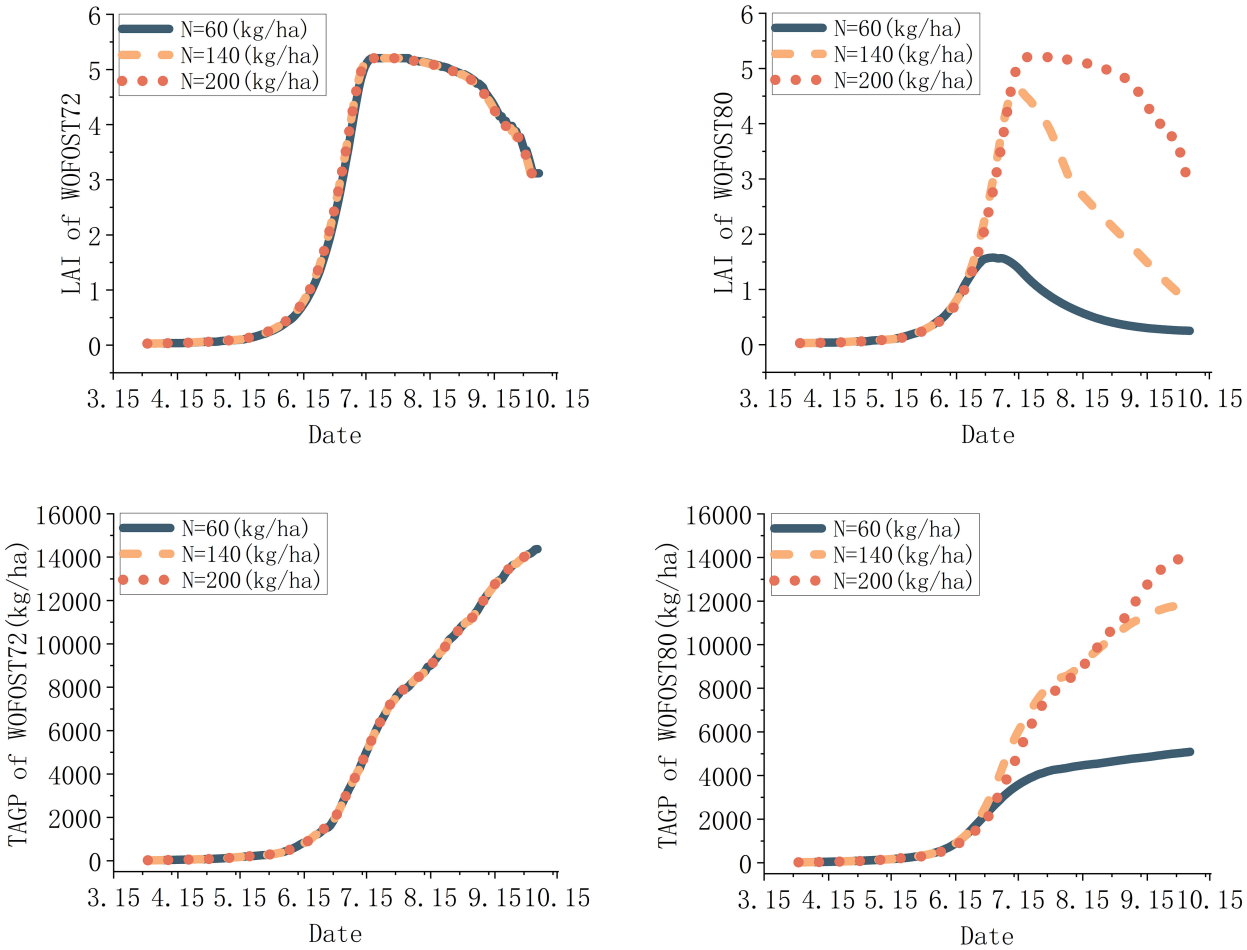
LAI and TAGP output of different versions of WOFOST under different nitrogen conditions.

The study first compares the results of two versions of the WOFOST model with different fertilization strategies in simulating crop growth and development. Simulate input parameters using the same agricultural management, crop parameters, soil parameters, scene parameters, and weather parameters, only changing the nitrogen application rate in the fertilization strategy. Based on the local standard nitrogen-based fertilizer application level of 200 kg/ha in the experimental field, reduce it by 30% and 70% respectively, and apply it in one go. Observe the crop simulation situation. As shown in **Figure 9**, the output results of the model indicate that the simulation of crop growth and development process by the WOFOST72 model is less affected by fertilization strategies, while WOFOST80 can significantly affect crop growth and development process through fertilization strategies. Based on the aim of this study to investigate the impact of fertilization strategies on rice, the WOFOST80 version was selected to simulate the growth and development process of rice, and fertilization decisions were made by assimilating aboveground biomass.

**Figure 10:**
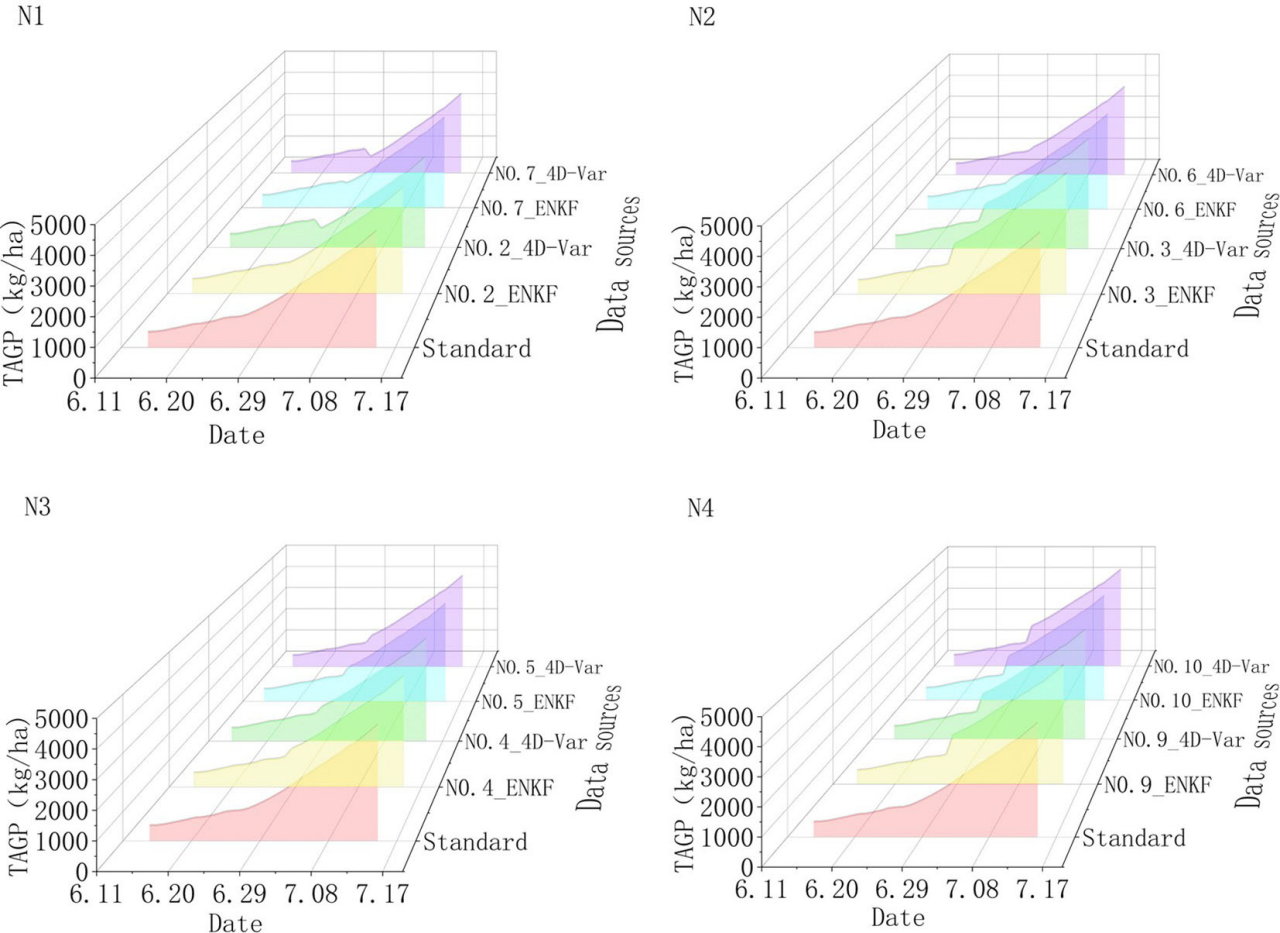
The assimilation results of WOFOST and inverted biomass. The vertical axis represents aboveground biomass (output parameter TAGP in the WOFOST model).

The assimilation localization model outputs aboveground biomass and inverted biomass from the unmanned aerial vehicle canopy hyperspectral data on June 28, 2021, to obtain the assimilated biomass. Except for control groups 1 and 8 experimental fields, the assimilated biomass of the other groups is shown in **Figure 10**. From the perspective of the fertilization gradient of base fertilizer, the observed biomass values in each field are consistent with the fertilization gradient of base fertilizer. Among them, fields 4 and 5 have a standard nitrogen fertilization gradient N3, and the observed values are close to the output values of the model. After assimilation, the results are consistent with the simulation results of the model; Fields 9 and 10 have a high nitrogen fertilization gradient N4, and their assimilated biomass results are higher than the standard situation. Fields 2 and 7 have a low nitrogen fertilization gradient N1, and its assimilated biomass results are lower than the standard situation with significant differences. Fields 3 and 6 have a low nitrogen fertilization gradient N2, with the assimilation results slightly lower than the standard situation and consistent with the bottom fertilizer gradient. The assimilation results of field 3 are higher than the standard situation and have a similar trend to the N4 gradient assimilation results. The reason may be that the soil nutrient content is higher, and rice absorbs more nutrients in the early stage and grows better. From the perspective of the assimilation effect, the assimilated biomass assimilation results of the 4D-Var algorithm are lower than those of the Ensemble Kalman Filter algorithm (ENKF), especially in fields 2 and 7 with a low nitrogen gradient N1 for fertilization. Overall, the assimilation results simulated the growth of rice in the experimental field well, providing an accurate data source for making fertilization decisions based on the simulation results of the rice growth process in the future.

### 3.3 Analysis of rice topdressing results based on simulation results of rice growth process

**Figure 11:**
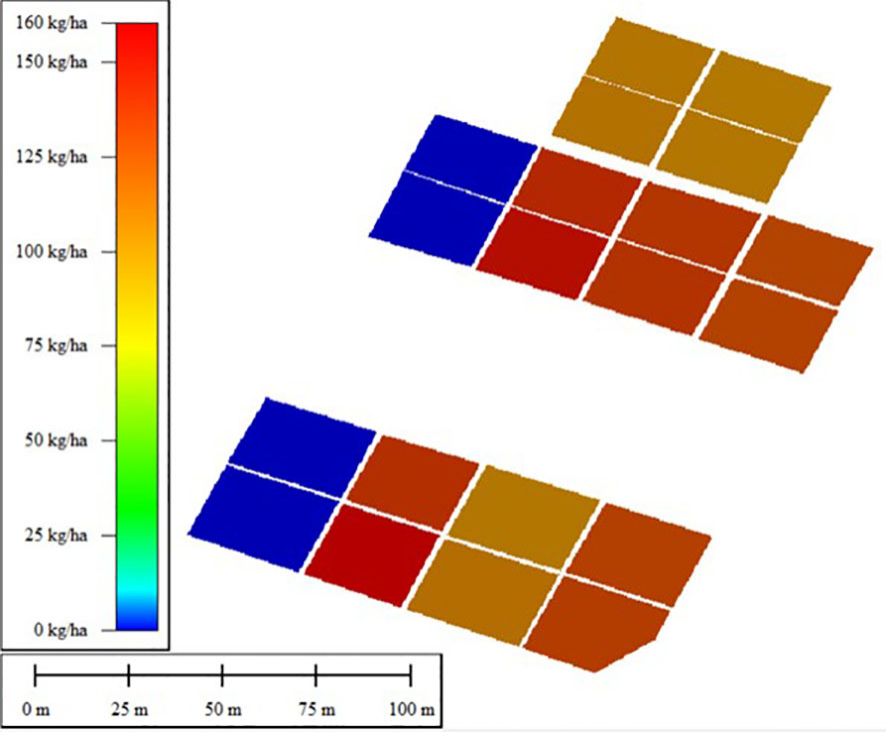
Prescription diagram for top dressing of plant protection drone operation (urea application for tillering fertilizer, with nitrogen content of 46%).

**Figure 12:**
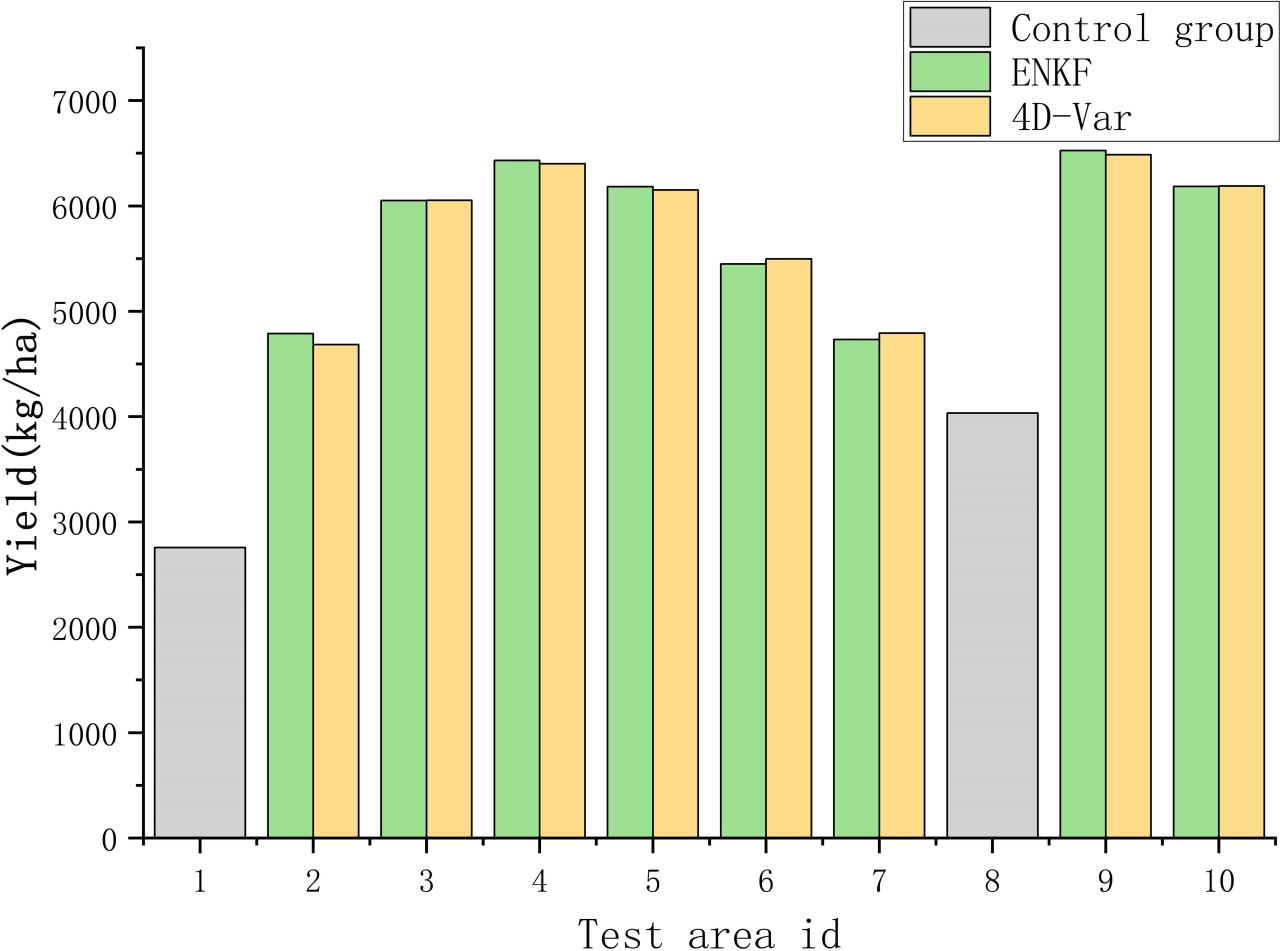
Bar chart of unit yield in each experimental community.

**Figure 13:**
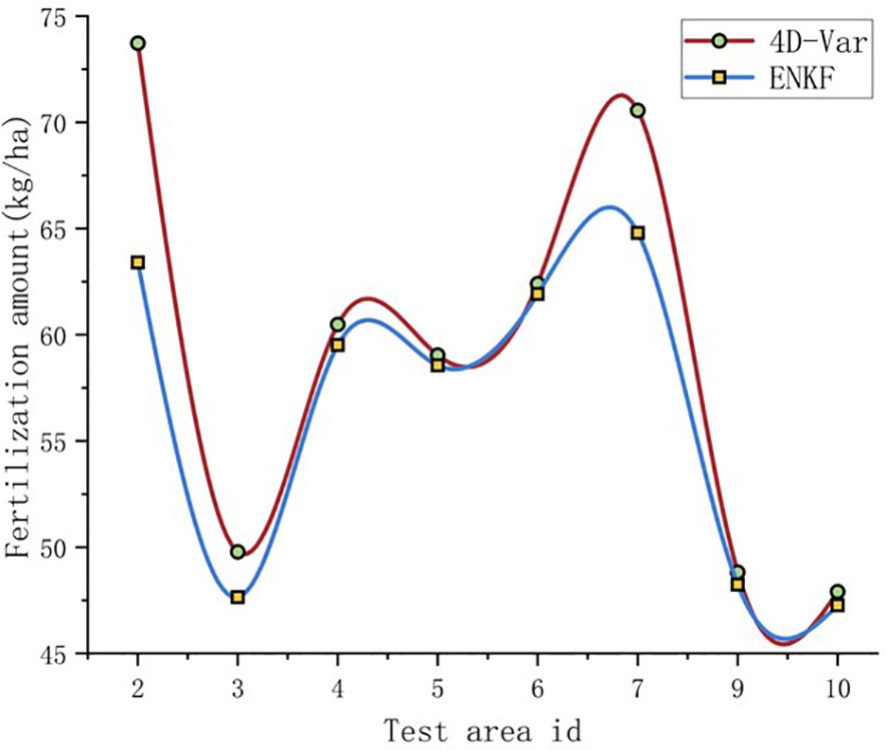
Fertilization amount during tillering stage (pure nitrogen).

This study used the fertilization amount of rice in the experimental field as the standard, and included three rounds of fertilization: base fertilizer, tillering fertilizer, and panicle fertilizer. The base fertilizer is applied according to the fertilization gradient, and the ear fertilizer is applied according to the standard fertilization scheme. This study mainly focuses on precise fertilization of tillering fertilizer. Each experimental plot is divided into two equal parts, and fertilization decisions are made based on the assimilated biomass results obtained from the Ensemble Kalman Filter (ENKF) algorithm and the Four Dimensional Variational Algorithm (4D-Var) algorithm. The fertilization prescription for plant protection drone fertilization is shown in **Figure 11**, which is formulated based on the fertilization decisions. The unit yield and fertilization situation of each experimental field are shown in **Figures 12**, **13**.

From the perspective of yield, the yield of experimental fields 1 and 8 based on the N0 fertilization gradient is significantly lower than the standard gradient yield. Among them, the lower yield of field 1 compared to field 8 is due to the occurrence of serious diseases. Except for fields 2 and 7 based on the N1 fertilization gradient, the overall yield of all other fertilization gradients tends to be consistent and the difference is small. Fields 4 and 5 based on standard fertilization gradient N3 have higher yields than other fertilization gradients, indicating that the standard fertilizer quantity is reasonable. Based on the N2 fertilization gradient, the fertilization gradients in fields 3 and 6 are consistent but there is a significant difference in yield. The higher yield in field 3 is consistent with the trend during assimilation. The reason for this may be related to factors such as higher soil nutrient content in field 3, which leads to more nutrients absorbed by rice in the early stage and better growth. The overall yield is lower than the average crop yield, which may be due to differences in meteorological data compared to the average weather data of the past three years.

From the perspective of fertilization amount, compared to the ENKF, the fertilization amount formulated based on the assimilation results of the 4D-Var method is higher, and the yield indicates that the yield of fertilization based on the two assimilation algorithms is the same. Therefore, when the rice yield reaches a consistent level, the required fertilization amount based on the ENKF fertilization strategy is lower, which is more in line with the requirements of economic and reducing nitrogen fertilizer use in the rice production process.

## 4 Discussion

### 4.1 Rice biomass inversion model based on hyperspectral data

In order to quickly obtain rice biomass information at the field scale and better quantify the growth of rice, this study collected hyperspectral data of unmanned aerial vehicle canopy and field experiment data that can reflect the biomass status of rice in 2020 as model inputs, combined with spectral processing and machine learning algorithms to obtain a rice biomass inversion model. In 2021, when conducting experiments to formulate fertilization decisions, this model was used to invert rice biomass as a state variable for data assimilation, achieving rapid and non-destructive monitoring of rice growth status.

The tillering stage is one of the peak nitrogen requirements for rice, but for row sown crops that have not yet been sealed, the overall biomass and leaf area index of rice plants in the early stage are relatively low, and the reflectance is easily submerged by interfering ground objects. Nutrient growth is crucial for the entire growth process and is also a focus of remote sensing monitoring. The hyperspectral data used to construct rice biomass inversion models during the tillering stage contains a large amount of redundant information. The next step is to introduce the mixed pixel decomposition method of hyperspectral images for satellite remote sensing images (Xusu, 2016; Yu et al., 2022 )into rice canopy spectra to remove factors such as water bodies, accurately obtain rice canopy spectral data, remove redundant information unrelated to biomass, and improve inversion efficiency.

The range of canopy spectra obtained by drones in this study is 400–1000nm. The large amount of original spectral data, severe collinearity between spectral wavelengths, and redundant information can lead to long model operation time and decreased classification accuracy. Using SPA to extract sensitive feature bands of biomass from preprocessed full band canopy hyperspectral data, selecting feature wavelengths that contain the most spectral information, the least redundant information, and the least collinearity to solve the above problems. When RMSECV reached its lowest value, seven characteristic bands sensitive to biomass in the canopy spectrum were selected.

Considering the complex characteristics of the spectra extracted by SPA, it may be difficult to establish a linear relationship between traditional statistical models and biomass. Therefore, this study takes the reflectance corresponding to the 7 feature bands selected by SPA as the model input, and rice biomass as the model output to construct an ELM based biomass inversion model. From the results, it can be seen that the constructed model can effectively estimate the biomass information of rice, better reflect the growth status of rice, and provide data support for subsequent data assimilation. This study only used one year of data to construct an inversion model and applied it in the second year. The amount of data is relatively small, and the training set can be expanded to improve the universality of the model in the future. ELM, as a machine learning algorithm based on feedforward neural networks, has the advantages of fast learning speed and strong convergence ability, but it also has the problem of easily falling into local optimal solutions. At present, research mainly focuses on optimizing algorithms for ELM (Liu et al., 2020), seeking better weight and bias configurations to improve the inversion accuracy and global search ability of algorithms.

### 4.2 Variable fertilization decision based on WOFOST model assimilation

Considering the advantages of the WOFOST model, which focuses on simulating annual field crops and has a relatively complete simulation of fertilizer, this study uses the WOFOST model to simulate the growth process of rice. In terms of WOFOST yield simulation, this study focuses on the differences in rice growth caused by nutrition, and other influencing factors such as disease and pest stress have not been considered. From the results, it can be seen that the output LAI and biomass of the localized model are consistent with the measured results, indicating the feasibility and accuracy of using parameter optimization algorithms to localize crop growth models. However, the accuracy of other output results of the model, such as the weight of each organ, needs to be verified in subsequent work.

Although the WOFOST model has strong mechanistic properties, it often requires a large number of parameters to be configured, and it is often used to simulate a region. To simulate the growth and development of fields at the field level, it is necessary to introduce remote sensing data with strong observation ability, and real-time performance, but lack of rationality to achieve the spatial heterogeneity of WOFOST model simulation results.

The final yield of each experimental plot was used as the evaluation criterion for fertilization decision-making. The experimental results showed that the yield of each experimental plot had the same trend as the fertilization gradient. For individual communities with significant differences in yield, the analysis may be influenced by differences in meteorological data input from the model and meteorological conditions in the experimental year, as well as input parameters such as soil fertility. The next step is to consider incorporating soil fertility into fertilization decision-making to improve the accuracy of model simulation and develop more accurate fertilization decisions. In terms of crop growth models, crop growth models such as ORYZA2000 that are suitable for simulating rice growth and development and have complete fertilization strategies can be attempted to make fertilization decisions. The experiment evaluates the advantages and disadvantages of each model in fertilization strategies using the same assimilation algorithm.

Using the fertilization amount in each experimental plot as the evaluation criterion for topdressing decision-making, the precise topdressing decision-making developed by this research method achieved relatively ideal yield results, and compared with traditional topdressing research that only relies on inversion data, it is more interpretable and solves the problem of lack of rationality. The experimental results show that the selection of standard fertilizer is reasonable, and the yield of each fertilization plot based on precise fertilization decision-making tends to be consistent. The experimental plot based on the fertilization gradient N1 has a low amount of bottom fertilizer application, and later fertilization cannot make up for the poor growth of rice in the early stage, so the final yield is lower than other experimental plots. The fertilization strategy formulated by the WOFOST model based on the Ensemble Kalman Filter algorithm to assimilate biomass under the same fertilization gradient reduces the average fertilization amount by 2.7kg/ha compared to the Four-Dimensional Variational Algorithm when obtaining the same yield. The next step is to compare the yield obtained from fertilization strategies formulated by multiple assimilation algorithms, and find more accurate assimilation algorithms for more fertilization strategies. In addition, multivariate assimilation can be combined with other data such as Leaf Area Index (LAI) and Soil moisture content (SM) to further improve the simulation accuracy of the model for actual situations and formulate more accurate fertilization decisions.

## 5 Conclusion

In order to accurately obtain the growth status of rice in each experimental plot, this study first constructed a hyperspectral inversion model for rice biomass. In 2020, field experiments were conducted to obtain rice growth process data. The hyperspectral data of rice canopy was used as input, and the continuous projection algorithm (SPA) was used to extract the hyperspectral features of rice canopy. Based on the ELM model, rice biomass inversion was implemented to achieve the goal of rapid acquisition of rice biomass data. The fertilization experiment was conducted in 2021, using ENKF and 4D-Var methods to assimilate and invert biomass results, and the localized WOFOST output aboveground biomass data to modify the WOFOFT model, so that the model can better simulate the growth status of each experimental plot. The output of the modified model is the simulated yield under the current fertilization scheme of the experimental plot. Using the WOFOST model to develop fertilization strategies and construct a prescription diagram for plant protection drone fertilization operations, with the average yield of the rice variety as the target, to achieve variable fertilization.

This study addresses the lack of mechanisms in traditional fertilization strategies based on unmanned aerial vehicle canopy hyperspectral data. By introducing remote sensing data, the WOFOST model simulation results have spatial heterogeneity, making it more suitable for simulating rice growth in small areas and formulating fertilization strategies. From the perspective of assimilation data, using aboveground biomass instead of previous studies using leaf area index (LAI) as assimilation data, and introducing the assimilation results into the decision-making of fertilization during the rice tillering stage, this is a new attempt.

The research results indicate that the assimilation results of both algorithms have achieved good assimilation effects, and the fertilization strategies formulated based on the two assimilation algorithms have achieved consistent yields compared to standard fertilization gradient fields. The fertilization strategy based on the ENKF assimilation method resulted in less fertilizer application and a 5.9% reduction compared to the standard fertilization scheme when achieving yields that were consistent with the fertilization strategy based on the 4D-Var assimilation method. Therefore, selecting reasonable biomass inversion methods, assimilation parameters, assimilation algorithms, and crop growth models is beneficial for the formulation of precision fertilization decisions.

## Data availability statement

The raw data supporting the conclusions of this article will be made available by the authors, without undue reservation.

## Author contributions

SL: Software, Visualization, Writing – original draft. ZJ: Conceptualization, Methodology, Writing – review & editing. JB: Formal analysis, Validation, Writing – review & editing. SX: Formal analysis, Software, Writing – review & editing. CX: Data curation, Investigation, Resources, Writing – review & editing. FY: Funding acquisition, Project administration, Supervision, Writing – review & editing.

## References

[B1] Amiri E. Irmak S. Yaghouti H. (2022). Performance of WOFOST model for simulating maize growth, leaf area index, biomass, grain yield, yield gap, and soil water under irrigation and rainfed conditions. J. Irrigation Drainage Eng. 148, 05021005. doi: 10.1061/(ASCE)IR.1943-4774.0001644

[B2] Challinor A. J. Wheeler T. R. Slingo J. M. Hemming D. (2005). Quantification of physical and biological uncertainty in the simulation of the yield of a tropical crop using present-day and doubled CO2 climates. Philos. Trans. R. Soc. London. Ser. B Biol. Sci. 360, 2085–2094. doi: 10.1098/rstb.2005.1740 16433095 PMC1569570

[B3] Charney J. G. Halem M. Jastrow R. J. J. A. (1969). Use of incomplete historical data to infer the present state of the atmosphere. J Atmos Sci. 26, 1160–1163. doi: 10.1175/1520-0469(1969)026<1160:UOIHDT>2.0.CO;2

[B4] Chen Y. Tao F. L. (2020). Improving the practicability of remote sensing data-assimilation-based crop yield estimations over a large area using a spatial assimilation algorithm and ensemble assimilation strategies. Agric. For. Meteorology 291, 108082. doi: 10.1016/j.agrformet.2020.108082

[B5] Cong C. Guangqiao C. Yibai L. Dong L. Bin M. Jinlong Z.. (2022). Research on monitoring methods for the appropriate rice harvest period based on multispectral remote sensing. Discrete Dynamics Nat. Soc. 2022, 1519667. doi: 10.1155/2022/1519667

[B6] Deepika P. Kaliraj S. (2021). “A survey on pest and disease monitoring of crops,” in International Conference on Signal Processing and Communications SPCOM. (Coimbatore, INDIA: IEEE).

[B7] Deng B. H. Zhang X. M. Gong W. Y. Shang D. P. (2019). “An overview of extreme learning machine,” in 4th International Conference on Control, Robotics and Cybernetics (CRC)). (Tokyo, JAPAN: IEEE), 189–195.

[B8] Dewenam L. E. F. Er-Raki S. Ezzahar J. Chehbouni A. (2021). Performance evaluation of the WOFOST model for estimating evapotranspiration, soil water content, grain yield and total above-ground biomass of winter wheat in Tensift Al Haouz (Morocco): application to yield gap estimation. Agronomy-Basel 11, 2480. doi: 10.3390/agronomy11122480

[B9] de Wit A. Boogaard H. Fumagalli D. Janssen S. Knapen R. Van Kraalingen D.. (2019). 25 years of the WOFOST cropping systems model. Agric. Syst. 168, 154–167. doi: 10.1016/j.agsy.2018.06.018

[B10] Dong Y. Y. Zhao C. J. Yang G. J. Chen L. P. Wang J. H. Feng H. K. (2013). Integrating a very fast simulated annealing optimization algorithm for crop leaf area index variational assimilation. Math. Comput. Model. 58, 871–879. doi: 10.1016/j.mcm.2012.12.013

[B11] Grooms I. (2022). A comparison of nonlinear extensions to the ensemble Kalman filter Gaussian anamorphosis and two-step ensemble filters. Comput. Geosciences 26, 633–650. doi: 10.1007/s10596-022-10141-x PMC8897550 35280324

[B12] He Z. Chen H. Liang L. Dong J. Liang Z. Zhao L. (2019). Alteration of crop rotation in continuous Pinellia ternate cropping soils profiled via fungal ITS amplicon sequencing. Lett. Appl. Microbiol. 68, 522–529. doi: 10.1111/lam.13135 30776140

[B13] Jia Y. J. Zhang H. J. Zhang X. Y. Su Z. B. (2022). Quantitative analysis and hyperspectral remote sensing inversion of rice canopy spad in a cold region. Engenharia Agricola 42, 42. doi: 10.1590/1809-4430-eng.agric.v42n4e20220030/2022

[B14] Ju J. Y. Lv Z. Y. Weng W. X. Zou Z. F. Lin T. H. Liu Y. Y.. (2023). A method for determining the nitrogen content of wheat leaves using multi-source spectral data and a convolution neural network. Agronomy-Basel 13, 2387. doi: 10.3390/agronomy13092387

[B15] Kärcher M. Boyaval S. Grepl M. A. Veroy K. (2018). Reduced basis approximation and a posteriori error bounds for 4D-Var data assimilation. Optimization Eng. 19, 663–695. doi: 10.1007/s11081-018-9389-2

[B16] Kumar U. Morel J. Bergkvist G. Palosuo T. Gustavsson A. M. Peake A.. (2021). Comparative analysis of phenology algorithms of the spring barley model in APSIM 7.9 and APSIM next generation: A case study for high latitudes. Plants-Basel 10, 443. doi: 10.3390/plants10030443 33652737 PMC7996762

[B17] Lai J. K. Lin W. S. (2021). Assessment of the rice panicle initiation by using NDVI-based vegetation indexes. Appl. Sciences-Basel 11, 10076. doi: 10.3390/app112110076

[B18] Li X. Du H. Mao F. Zhou G. Han N. Xu X.. (2019). Assimilating spatiotemporal MODIS LAI data with a particle filter algorithm for improving carbon cycle simulations for bamboo forest ecosystemsyy. Sci. Total Environ. 694, 133803. doi: 10.1016/j.scitotenv.2019.133803 31756841

[B19] Li R. Li C. J. Dong Y. Y. Liu F. Wang J. H. Yang X. D.. (2011). Assimilation of remote sensing and crop model for LAI estimation based on ensemble Kalman filter. Agric. Sci. China 10, 1595–1602. doi: 10.1016/S1671-2927(11)60156-9

[B20] Li G. S. Wu D. H. Su Y. C. Kuo B. E. Yang M. E. Lai M. H.. (2021). Prediction of plant nutrition state of rice under water-saving cultivation and panicle fertilization application decision making. Agronomy-Basel 11, 1626. doi: 10.3390/agronomy11081626

[B21] Liu T. Fan Q. Kang Q. Niu L. (2020). Extreme learning machine based on firefly adaptive flower pollination algorithm optimization. Processes 8, 1583. doi: 10.3390/pr8121583

[B22] Liu J. Hou X. Chen S. Mu Y. Huang H. Wang H.. (2023b). A method for estimating yield of maize inbred lines by assimilating WOFOST model with Sentinel-2 satellite data. Front. Plant Sci. 14, 1201179. doi: 10.3389/fpls.2023.1201179 37746025 PMC10513754

[B23] Liu H. Lei X. Liang H. Wang X. (2023a). Multi-model rice canopy chlorophyll content inversion based on UAV hyperspectral images. Sustainability 15, 7038. doi: 10.3390/su15097038

[B24] Lu J. S. Li W. Y. Yu M. L. Zhang X. B. Ma Y. Su X.. (2021). Estimation of rice plant potassium accumulation based on non-negative matrix factorization using hyperspectral reflectance. Precis. Agric. 22, 51–74. doi: 10.1007/s11119-020-09729-z

[B25] Lu B. H. Yu K. Wang Z. M. Wang J. Mao L. J. (2019). “Preliminary approach on adaptability of ORYZA2000 model for single cropping rice in Jiangsu province (China),” in International Conference on Agro-Geoinformatics. (Istanbul, TURKEY: IEEE), 1–5.

[B26] Luo S. J. Jiang X. Q. Jiao W. H. Yang K. L. Li Y. J. Fang S. H. (2022). Remotely sensed prediction of rice yield at different growth durations using UAV multispectral imagery. Agriculture-Basel 12, 1447. doi: 10.3390/agriculture12091447

[B27] Lv Z. F. Zhu Y. Liu X. J. Ye H. B. Tian Y. C. Li F. F. (2018). Climate change impacts on regional rice production in China. Climatic Change 147, 523–537. doi: 10.1007/s10584-018-2151-0

[B28] Ma H. J. Malone R. W. Jiang T. C. Yao N. Chen S. Song L. B.. (2020). Estimating crop genetic parameters for DSSAT with modified PEST software. Eur. J. Agron. 115, 126017. doi: 10.1016/j.eja.2020.126017

[B29] Pique G. Fieuzal R. Ceschia E. (2020). “Estimation of biomass and CO2 fluxes of sunflower by assimilating Hstr data in a simple crop model,” in Mediterranean and Middle-East Geoscience and Remote Sensing Symposium (M2GARSS)). (Tunis, TUNISIA: IEEE), 318–321.

[B30] Pu Z. X. Hacker J. (2009). Ensemble-based Kalman filters in strongly nonlinear dynamics. Adv. Atmospheric Sci. 26, 373–380. doi: 10.1007/s00376-009-0373-9

[B31] Storto A. Tveter F. T. (2009). Assimilating humidity pseudo-observations derived from the cloud profiling radar aboard CloudSat in ALADIN 3D-Var. Meteorological Appl. 16, 461–479. doi: 10.1002/met.144

[B32] Tang Q. Mu L. J. Sidorenko D. Goessling H. Semmler T. Nerger L. (2020). Improving the ocean and atmosphere in a coupled ocean-atmosphere model by assimilating satellite sea-surface temperature and subsurface profile data. Q. J. R. Meteorological Soc. 146, 4014–4029. doi: 10.1002/qj.3885

[B33] Tang R. Supit I. Hutjes R. Zhang F. Wang X. Chen X.. (2023). Modelling growth of chili pepper (Capsicum annuum L.) with the WOFOST model. Agric. Syst. 209, 103688. doi: 10.1016/j.agsy.2023.103688

[B34] Wang P. C. Gao B. Gong X. Tong L. Sun Y. Gu X. F. (2020). “The research of leaf area index analyzer based on embedded platform,” in IEEE International Symposium on Geoscience and Remote Sensing IGARSS. (ELECTR NETWORK: IEEE), 4311–4314.

[B35] Wang D. L. Li R. Liu T. Liu S. P. Sun C. M. Guo W. S. (2023). Combining vegetation, color, and texture indices with hyperspectral parameters using machine-learning methods to estimate nitrogen concentration in rice stems and leaves. Field Crops Res. 304, 109175. doi: 10.1016/j.fcr.2023.109175

[B36] Wang X. D. Wang Y. L. Zhang Y. P. Xiang J. Zhang Y. K. Zhu D. F.. (2021). The nitrogen topdressing mode of indica-japonica and indica hybrid rice are different after side-deep fertilization with machine transplanting. Sci. Rep. 11, 1494. doi: 10.1038/s41598-021-81295-4 33452412 PMC7810741

[B37] Xu S. Z. Xu X. A. Blacker C. Gaulton R. Zhu Q. Z. Yang M.. (2023). Estimation of leaf nitrogen content in rice using vegetation indices and feature variable optimization with information fusion of multiple-sensor images from UAV. Remote Sens. 15, 854. doi: 10.3390/rs15030854

[B38] Xusu (2016). “Compressive sensing for endmember extraction,” in 2nd IEEE International Conference on Computer and Communications (ICCC)). (Chengdu, PEOPLES R CHINA: IEEE), 1345–1348.

[B39] Yu F. H. Zhao D. Guo Z. H. Jin Z. Y. Guo S. Chen C. L.. (2022). Characteristic analysis and decomposition of mixed pixels from UAV hyperspectral images in rice tillering stage. Spectrosc. Spectral Anal. 42, 947–953. doi: 10.3964/j.issn.1000-0593(2022)03-0947-07

[B40] Zhang J. Y. Zhu J. F. Yang L. Y. Li Y. L. Wang W. R. Zhou X. R.. (2023). Mapping of the Waxy Gene in Brassica napus L. via Bulked Segregant Analysis (BSA) and Whole-Genome Resequencing. Agronomy-Basel 13, 2611. doi: 10.3390/agronomy13102611

[B41] Zhou H. K. Geng G. P. Yang J. H. Hu H. Sheng L. Lou W. D. (2022). Improving Soil Moisture Estimation via Assimilation of Remote Sensing Product into the DSSAT Crop Model and Its Effect on Agricultural Drought Monitoring. Remote Sens. 14, 3187. doi: 10.3390/rs14133187

[B42] Zhou G. Liu M. Liu X. Li J. (2018). “Combination of crop growth model and radiation transfer model with remote sensing data assimilation for fapar estimation,” in IEEE International Symposium on Geoscience and Remote Sensing IGARSS. (Valencia, SPAIN: IEEE), 1882–1885.

